# PPARs: Regulators and Translational Targets in the Lung

**DOI:** 10.1155/2012/342924

**Published:** 2012-11-04

**Authors:** Raju C. Reddy, Virender K. Rehan, Jesse Roman, Patricia J. Sime

**Affiliations:** ^1^Division of Pulmonary, Allergy and Critical Care Medicine, Department of Medicine, Emory University and Atlanta VA Medical Center, Atlanta, GA 30033, USA; ^2^Department of Pediatrics, Los Angeles Biomedical Research Institute, Harbor-UCLA Medical Center and David Geffen School of Medicine, University of California, Los Angeles, Torrance, CA 90502, USA; ^3^Division of Pulmonary, Critical Care and Sleep Disorders, Department of Medicine, University of Louisville School of Medicine, Louisville, KY 40202, USA; ^4^Robley Rex Veterans Affairs Medical Center, Louisville, KY 40202, USA; ^5^Division of Pulmonary and Critical Care, Department of Medicine, University of Rochester School of Medicine and Dentistry, Box 692, Rochester, NY 14642, USA

In this special issue, we present a selection of review articles and new studies that cover many aspects of PPAR biology and their potential translational implications. These papers provide cogent examples of the importance of PPARs in the lung and its diseases, focusing mainly on PPAR-*γ*. They illustrate the wide variety of functions served by this receptor and indeed all PPARs. 

 For example, PPAR-*γ* in epithelial cells is essential for normal development of fetal and neonatal lungs, suggesting that PPAR-*γ* agonists might be useful for treating bronchopulmonary dysplasia of prematurity and similar conditions. Another prominent role of PPAR-*γ* and other PPARs is regulation of inflammatory responses. The influence of PPARs on inflammation is exerted through many different pathways, including effects on migration of inflammatory cells from the bloodstream into affected tissues. Effects of PPARs on eosinophils are somewhat complex, however, as high concentrations of PPAR-*γ* activators inhibit migration while low concentrations stimulate it, probably by upregulating non-directional cell movement (chemokinesis) rather than directed chemotaxis. PPAR-*γ* is also a key regulator of fibroblast transdifferentiation, promoting differentiation into adipocytes while inhibiting transformation of fibroblasts to myofibroblasts. These findings suggest that PPAR-*γ* agonists might have therapeutic utility for fibrosis. Interestingly, new findings provide evidence that intracellular mycobacterial pathogens including *M. tuberculosis *can activate PPAR-*γ*, exploiting its immunosuppressive actions to subvert the immune response while promoting an intracellular environment favorable for mycobacterial survival by stimulating accumulation of lipid droplets. 

Early research identified PPAR-*α* and PPAR-*γ* as metabolic regulators, but later discoveries revealed far broader regulatory roles for all three PPARs. Their anti-inflammatory roles may be particularly important in the lung, which is constantly exposed to infectious agents and noxious stimuli and yet depends on the integrity of delicate structures such as the alveolar-capillary interface for its crucial gas-exchange function. All three PPARs exert anti-inflammatory effects and are widely distributed in the lung. PPAR-*γ* in particular is highly expressed in lung epithelium and endothelium as well as in alveolar macrophages, acting in these cells to limit inflammation and promote its resolution. Activated PPARs act through several mechanisms, including transrepression of proinflammatory transcription factors such as NF-*κ*B. In this way, they limit the production of cytokines and other mediators that drive inflammation. Together, these findings point to potential utility of PPARs as targets for treatment of selected inflammatory lung diseases. 

A key challenge is to discover and clearly define the roles of endogenous PPAR agonists, and whether inadequate upregulation of PPAR activity by endogenous agonists contributes to pathogenesis of certain diseases. No endogenous ligands are currently known for PPAR-*β*/*δ*, but recent discoveries demonstrated two distinct endogenous PPAR-*α* agonists that are physiologically relevant in different cell types: a specific phospholipid in hepatocytes and leukotriene B_4_ in cells of the immune system. Such utilization of different PPAR-*α* agonists presumably permits regulation by molecules relevant to key functions of the respective cells (phospholipids in hepatocytic lipid metabolism; leukotriene B_4_ in immune cell responses to infection and inflammatory stimuli). Cell-type selective effects of relevant PPAR agonists represent a potentially important mechanism for pleiotropic physiological regulation via common and widely distributed receptors. It will be interesting to discover whether other PPARs follow a similar pattern.

The search for endogenous ligands of PPAR-*γ* has been a major focus of research. Several candidates have been identified, although the physiological relevance of most of them remains questionable. Perhaps the strongest current candidates are nitrated fatty acids (NFAs), which are produced by nonenzymatic reactions of NO (and its products) with unsaturated fatty acids. NFAs activate all three PPARs, but are most potent for PPAR-*γ*. NO production is often upregulated during inflammation, which would tend to raise local NFA concentrations and thereby trigger anti-inflammatory effects of PPAR activation, thus presumably mitigating inflammatory tissue damage. The total concentrations of NFAs in the bloodstream significantly exceed their EC_50_ for PPAR-*γ* activation. However, most circulating NFAs are either esterified or bound to plasma proteins. A portion of the esterified NFAs could be released due to inflammation-induced upregulation of cholesterol ester hydrolases, thus providing a further basis for physiological, feedback-driven inflammatory modulation by NFAs. Nevertheless, whether concentrations of free endogenous NFAs are sufficient for efficacious PPAR-*γ* activation remains unknown. Furthermore, like PPAR-*α*, PPAR-*γ* may prove to utilize different endogenous agonists for different, functionally diverse, cell types in which it is expressed. Accordingly, efforts are underway to develop synthetic PPAR-*γ* agonists that effectively activate it in a targeted, cell type-selective manner. Successful development of such ligands could reduce the likelihood of unwanted effects on non-target tissues.

Synthetic PPAR agonists, both novel and established, are a focus of translational and therapeutic interest, although many of these have been found to also exert significant PPAR-independent effects. Recent years have seen the development, although not yet the clinical use, of “triple” agonists that activate all three PPARs. The PPAR-*γ*-activating thiazolidinediones and PPAR-*α*-activating fibrates alike have been used clinically for well over a decade. Some of these drugs can cause adverse effects during long-term use, such as cardiovascular events that led to restrictions on rosiglitazone use. However, such adverse effects might be reduced by shorter periods of use, or by using lower doses made possible by synergy with other therapeutic agents. Preclinical studies have demonstrated such synergy in several diseases including certain cancers. In this connection, a preliminary clinical study of pioglitazone as potential neoadjuvant therapy in non-small-cell lung cancer is currently underway, while two phase I studies of a novel PPAR-*γ* agonist in combination with the established chemotherapeutic agents carboplatin/paclitaxel or erlotinib in patients with non-small-cell lung cancer have recently been completed.

In asthma, preclinical studies have strongly supported the beneficial effects of PPAR-*γ* agonists. Two studies of pioglitazone as asthma therapy are currently in progress, one of which is restricted to patients whose severe asthma is not adequately controlled by currently available treatments. Such patients would clearly benefit if pioglitazone or other PPAR-*γ* agonists prove helpful. There are no clinical trials of PPAR-*γ* agonists in other pulmonary diseases. However, considering available preclinical evidence for the benefits and relative safety of these drugs, along with the current absence of satisfactory therapy in many instances, it would be reasonable to conduct such trials in diseases such as COPD. It is also likely that we will see clinical trials of PPAR-*β*/*δ* agonists, which to date have not found therapeutic application.

In clinical trials, as in current clinical practice, PPAR agonists are administered orally. A promising alternative delivery route for treatment of lung diseases is via inhalation, which is advantageous because it achieves maximum drug concentrations at the intended target while limiting systemic side effects. Inhalation can also circumvent problems pertaining to oral bioavailability and first-pass metabolism. This is already the usual route of administration for anti-inflammatory glucocorticoids in treatment of asthma and other lung diseases, as it minimizes systemic adverse effects. There is currently no inhalable formulation of any PPAR agonist, but development of such formulations seems feasible and might enhance the range of therapeutic applications of PPAR agonists in lung disease.

We appreciate the opportunity to share exciting research on the varied roles that PPARs play in lung biology and disease and hope that the papers presented here will stimulate further important research and translational progress.

*Raju C. Reddy* *Raju C. Reddy* *Virender K. Rehan* *Virender K. Rehan* *Jesse Roman* *Jesse Roman* *Patricia J. Sime* *Patricia J. Sime*

